# A Deep-Learning Approach for Inference of Selective Sweeps from the Ancestral Recombination Graph

**DOI:** 10.1093/molbev/msab332

**Published:** 2021-11-22

**Authors:** Hussein A Hejase, Ziyi Mo, Leonardo Campagna, Adam Siepel

**Affiliations:** 1 Simons Center for Quantitative Biology, Cold Spring Harbor Laboratory, Cold Spring Harbor, NY, USA; 2 School of Biological Sciences, Cold Spring Harbor Laboratory, Cold Spring Harbor, NY, USA; 3 Fuller Evolutionary Biology Program, Cornell Lab of Ornithology, Ithaca, NY, USA; 4 Department of Ecology and Evolutionary Biology, Cornell University, Ithaca, NY, USA

**Keywords:** ancestral recombination graph, machine learning, positive selection, selective sweep

## Abstract

Detecting signals of selection from genomic data is a central problem in population genetics. Coupling the rich information in the ancestral recombination graph (ARG) with a powerful and scalable deep-learning framework, we developed a novel method to detect and quantify positive selection: **S**election **I**nference using the **A**ncestral recombination graph (SIA). Built on a Long Short-Term Memory (LSTM) architecture, a particular type of a Recurrent Neural Network (RNN), SIA can be trained to explicitly infer a full range of selection coefficients, as well as the allele frequency trajectory and time of selection onset. We benchmarked SIA extensively on simulations under a European human demographic model, and found that it performs as well or better as some of the best available methods, including state-of-the-art machine-learning and ARG-based methods. In addition, we used SIA to estimate selection coefficients at several loci associated with human phenotypes of interest. SIA detected novel signals of selection particular to the European (CEU) population at the *MC1R* and *ABCC11* loci. In addition, it recapitulated signals of selection at the *LCT* locus and several pigmentation-related genes. Finally, we reanalyzed polymorphism data of a collection of recently radiated southern capuchino seedeater taxa in the genus *Sporophila* to quantify the strength of selection and improved the power of our previous methods to detect partial soft sweeps. Overall, SIA uses deep learning to leverage the ARG and thereby provides new insight into how selective sweeps shape genomic diversity.

## Introduction

The ability to accurately detect and quantify the influence of selection from genomic sequence data enables a wide variety of insights, ranging from understanding historical evolutionary events to characterizing the functional and disease relevance of observed or potential genetic variants. Adaptive evolution is driven by increases in frequency of alleles that enhance reproductive fitness. In addition, alleles experiencing such positive selection often provide insights into the functional or mechanistic basis of phenotypes of interest. Examples of genetic determinants of important phenotypic traits under selection in human populations include a family of mutations in the hemoglobin-β cluster, which confer resistance to malaria and are at high frequencies in many populations (Currat et al. 2002; Ohashi et al. 2004), loci controlling growth factor signaling pathways that contribute to short stature in Western Central African hunter-gatherer populations (Jarvis et al. 2012; Lachance et al. 2012), as well as mutations in several genes involved in immunity, hair follicle development, and skin pigmentation (Sabeti et al. 2007) (reviewed in Sabeti et al. [2006]; Kelley and Swanson [2008]; Fu and Akey [2013]; Hejase, Dukler, et al. [2020]).

Population genetic methods predominantly identify positive selection through the detection of selective sweeps. As the frequency of an advantageous allele increases, linked variants in the vicinity can “hitchhike” to high frequency, leading to local reductions in genetic diversity. Previous approaches to detecting selective sweeps (such as traditional summary statistics [Tajima 1989], approximate likelihood and Approximate Bayesian Computation [ABC] methods [Peter et al. 2012], or supervised machine-learning [ML] methods [Schrider and Kern 2016; Kern and Schrider 2018]) exploit the effect of genetic hitchhiking on the spatial haplotype structure and site frequency spectrum (SFS). Summary statistics have the advantage of being fast and easy to compute, but may confound the effects of selection on genetic diversity with the effects of complex demographic histories including bottlenecks, population expansions, and structured populations. Besides, they cannot easily be used to estimate the value of the selection coefficient. Approximate likelihood and ABC methods, on the other hand, can provide an estimate of the strength of selection by aggregating multiple summary statistics (Peter et al. 2012), but can be prohibitively computationally expensive when applied at a large scale. ML methods for inferring selection can be more scalable and can capture complex nonlinear relationships among features. With the exception of a handful of recently developed methods that operate on the multiple sequence alignment itself (Flagel et al. 2019; Torada et al. 2019), however, the majority of ML approaches to selection inference solely make use of traditional summary statistics as features for prediction. In short, previous methods (including ABC and most ML methods) predominantly rely on low-dimensional summary statistics, which, even in combination, capture only a small portion of the information in the sequence data.

Recently, a new generation of inference methods have made it possible to go beyond summary statistics and estimate or sample a full ancestral recombination graph (ARG) (Hudson 1990; Griffiths and Marjoram 1996; Wiuf and Hein 1999) for a collection of sequences of interest. The ARG is a complex data structure that summarizes the shared evolutionary history and recombination events that have occurred in a collection of DNA sequences, and therefore contains highly informative features that can potentially be leveraged to make accurate inferences about selection. The ARG representation is interchangeable with a sequence of local genealogies along the genome and the recombination events that transform each genealogy to the next. The influence of selection on each allele can be characterized from the ARG, based on departures from the patterns of coalescence and recombination expected under neutrality as reflected in the local genealogies. Traditional ARG inference methods (Hein 1993; Song and Hein 2005; Minichiello and Durbin 2006; Kuhner 2006; O’Fallon 2013) were restricted in accuracy and scalability, limiting the practical application of ARGs. Recent advances (Rasmussen et al. 2014), however, have enabled scalable yet statistically rigorous genome-wide ARG inference with dozens of genomes. Moreover, methods such as Relate (Speidel et al. 2019) and tsinfer (Kelleher et al. 2019) have further dramatically improved the scalability of ARG inference to accommodate thousands or even hundreds of thousands of genomes. The latest progress in genealogical inference has paved the way for ARG-based methods to address many different questions in population genetics (Arenas 2013; Rasmussen et al. 2014; Kelleher et al. 2019; Speidel et al. 2019).

One natural way to exploit the richness of the ARG representation in inference of selection would be to extract features from inferred ARGs and feed them into a modern supervised ML framework. Deep-learning methods, in particular, have recently achieved unprecedented success on a variety of challenging problems, including image recognition, machine translation, and game-play (LeCun et al. 2015). Deep learning is also highly flexible, providing many opportunities for the design of novel model architectures motivated by biological knowledge. An ARG-guided deep-learning model could potentially provide new insight into how natural selection impacts the human genome, human diseases and other phenotypes, and human evolution.

**Fig. 1.**
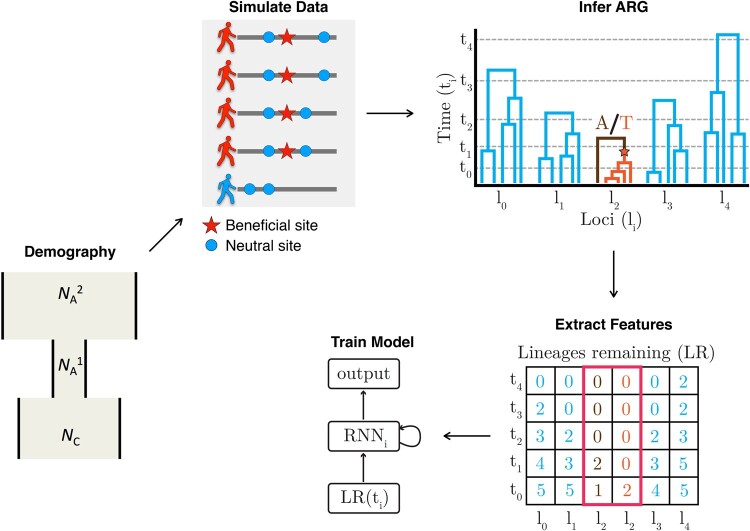
A high-level framework for automating the detection of selective sweeps. We first estimate the demographic history for the population of interest, then based on the estimated demographic history, we simulate neutral regions and sweeps using the discoal simulator (Kern and Schrider 2016). We proceed with ARG inference and then extract ARG-level statistics from each simulated region. The ARG-level statistics are used as features for a deep-learning RNN model. Finally, the trained model is applied to the empirical data to infer sweeps, selection coefficients, and AF trajectories.

With these goals in mind, we developed a new method, called SIA (**S**election **I**nference using the **A**ncestral recombination graph), that uses a Recurrent Neural Network (RNN) (Hochreiter and Schmidhuber 1997; Maas et al. 2011) to infer the selection coefficient and allele frequency (AF) trajectory of a variant that maps to a gene tree embedded in an ARG. Rather than relying on traditional sequence-based summary statistics, SIA makes use of features based on the local genealogies extracted from the ARG. Based on these local topological features, SIA learns to infer the selection coefficient and AF trajectory of a beneficial variant (see fig. 1). As described below, SIA performs well on benchmarks and is reasonably robust to model mis-specification. Applying SIA to data from the 1000 Genomes Northern and Western European (CEU) population, we identified new and known loci under positive selection that are associated with a variety of phenotypes and estimated selection coefficients at these loci. In addition, using SIA, we built on our previous work (Hejase, Salman-Minkov, et al. 2020) on a bird species-complex in the genus *Sporophila* by elucidating the strength and targets of selection at specific loci tied to a collection of rapid speciation events. Overall, SIA is the first method that couples ARG-based features with an ML approach for population genetic inference.

## Results

### Methodological Overview

SIA is based on an RNN that is trained to predict selection at a genomic site from genealogical features at that site of interest and nearby sites (see Materials and Methods for detailed descriptions; see fig. 1 for a conceptual overview of SIA; and supplementary fig. S1, Supplementary Material online for an illustration of ARG features and the RNN architecture). Based on the demography of a particular population of interest, training data including genomic regions under various strengths of selection are simulated. The ARG is then inferred from each simulated data set. ARG-level statistics are extracted at the site under selection (or a neutral site) as features to be used as input to the deep-learning model. Specifically, we use lineage counts at a set of discrete time points as a fixed-dimension encoding of a genealogy. The encoding of the genealogy at the focal site as well as similar encodings of flanking genealogies constitute the feature vector for that site. SIA uses a Long Short-Term Memory (LSTM) architecture, designed specifically to handle the temporal nature of the feature set. The LSTM unrolls temporally such that the lineage counts at each time point are fed to the network iteratively. Finally, the model trained on simulations is applied to ARGs inferred from empirical data to identify sweeps, infer selection coefficients, and AF trajectories.

### Classification of Sweeps

**Fig. 2.**
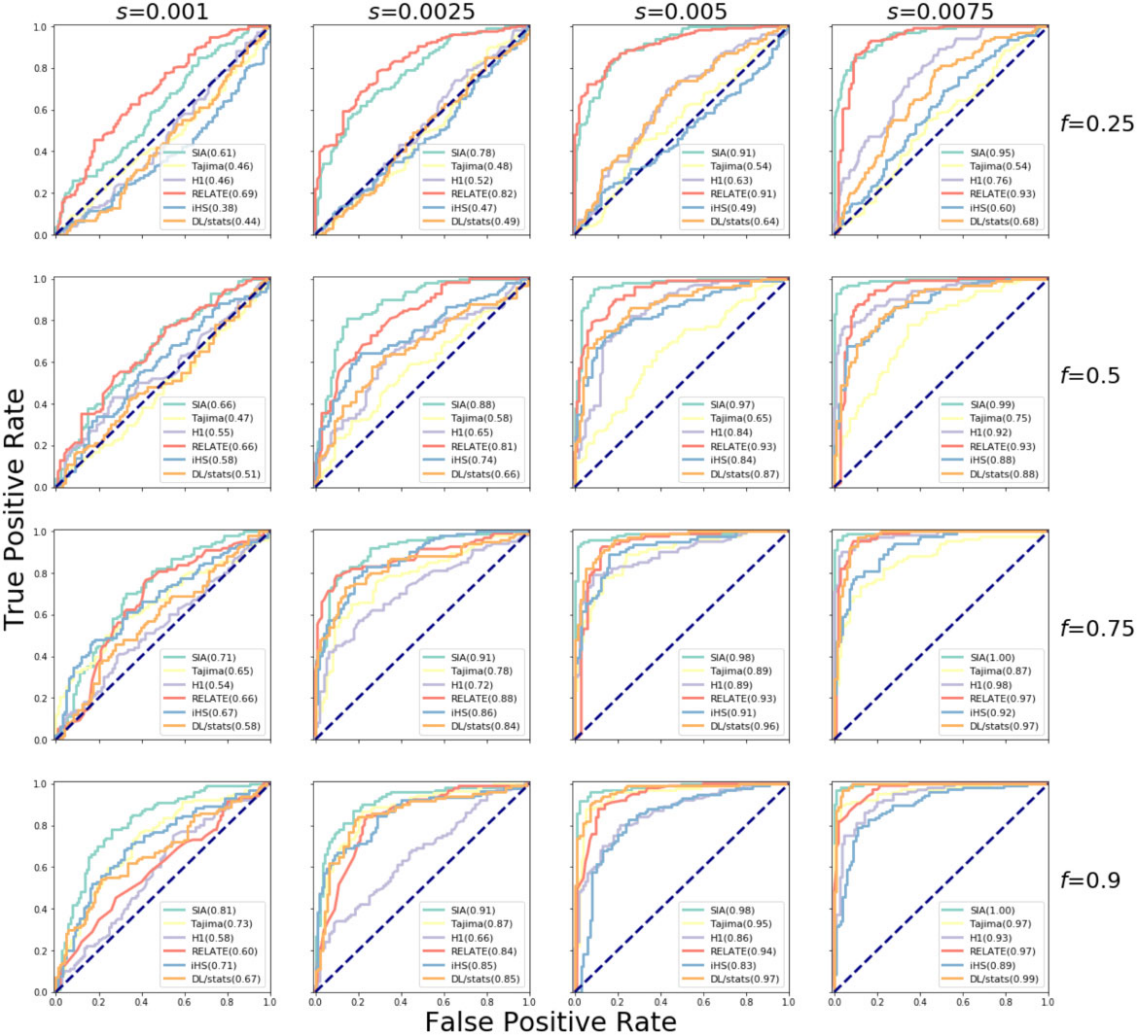
Classification performance of SIA and other methods on simulated data. Sequence data were simulated under a variety of selection regimes (*s*, shown horizontally) and DAFs for the beneficial mutation under selection (*f*, shown vertically) (see Materials and Methods for more details). The prediction task distinguished neutral regions and sweeps. The methods were tested on a set of 200 regions per panel (100 per class), and the ROC curve records the true positive rate (TPR) as a function of the false positive rate (FPR). The curve is obtained by varying the prediction threshold from 0 to 1 and recording for each threshold the number of regions correctly assigned (TPs) or misassigned (FPs) as positives (with prediction probability above the threshold). The performance of each method was evaluated based on the area under its ROC curve, or AUROC (shown in parenthesis in figure legend). Note that inferred genealogies were used as input to SIA.

We first compared SIA with several existing methods, including the Tajima’s D (Tajima 1989) and H1 (Garud et al. 2015) summary statistics, iHS (Voight et al. 2006), a genealogy-based statistic (Speidel et al. 2019), and a summary-statistic-based ML method (Schrider and Kern 2016; Kern and Schrider 2018) (see Materials and Methods), in the classification task of distinguishing hard sweeps from neutrally evolving regions. Our performance comparison was conducted across 16 combinations of selection coefficients and segregating allele frequencies such that the beneficial site was subjected to selection ranging from weak to strong, resulting in low to high derived allele frequencies (DAFs). Because a priori we expected sweep sites with lower selection coefficients and lower DAFs to be harder to detect, we performed a stratified analysis of SIA’s performance by selection coefficient and DAF. Figure 2 reports the receiver-operating characteristic (ROC) curves using simulations based on the CEU demographic model (Tennessen et al. 2012) where inferred genealogies were used as input to SIA to account for gene tree uncertainty. As expected, all methods tended to perform better in a regime with higher selection coefficients and DAFs, as indicated by increasing values of the area under the ROC curve (AUROC) statistic from left to right (increasing selection) and from top to bottom (increasing DAF). SIA outperformed the other methods across model conditions, with a more pronounced performance advantage for sites under weaker selection and segregating at lower DAFs (fig. 2). For each given selection coefficient, the AUROC of the Relate tree statistic (shown in red in fig. 2), which measures how unlikely it is that the observed expansion of the derived lineages is purely due to genetic drift, did not substantially improve as the DAF increased. Alleles at higher frequency tend to be older and subjected to drift over longer periods, which may lead to reduced power for Relate to distinguish lineage expansion under selection from the neutral expectation. Consequently, although the ARG-based methods SIA and Relate both outperformed other methods at low DAFs, SIA was alone in maintaining this advantage at higher DAFs.

In addition, we validated the ability of SIA to classify genomic regions with additional test sets simulated under a demographic model for southern capuchinos, a group of songbirds in which we previously identified and characterized many examples of sweeps (Hejase, Salman-Minkov, et al. 2020), finding a predominance of “soft” rather than “hard” sweeps (meaning that they tend to be based on standing genetic variation rather than new mutations; see Materials and Methods). Supplementary figure S2, Supplementary Material online reports the ROC curves for the task of distinguishing partial soft sweeps from neutral regions. Despite soft sweeps being harder to detect, the classifier achieved good performance in the moderate-to-strong selection regimes (*s* = 0.005 and *s* = 0.0075) where the accuracy ranged between 82% and 96%, a substantial improvement over the previous accuracy of 56% (Hejase, Salman-Minkov, et al. 2020). SIA performed particularly well in identifying partial soft sweeps when the site under selection was at a high segregating frequency. For example, at segregating frequencies of 0.75 and 0.9, the performance of SIA ranged between 80% and 96% across a variety of selection regimes (*s* = 0.0025, 0.005, and 0.0075). The performance of SIA degraded somewhat for weak selection (*s* = 0.001) with an accuracy ranging between 63% and 74%.

### Selection Coefficient Inference Using True Gene Trees

We assessed the performance of SIA in correctly predicting the selection coefficient and compared it with CLUES (Stern et al. 2019). Like SIA, CLUES uses local genealogies based on the ARG to infer a selection coefficient. However, CLUES calculates the likelihood of the genealogy analytically using a hidden Markov model (HMM), and does not rely on simulated training data. In addition, CLUES uses a single genealogy at the focal site, whereas SIA additionally considers flanking trees.

**Fig. 3.**
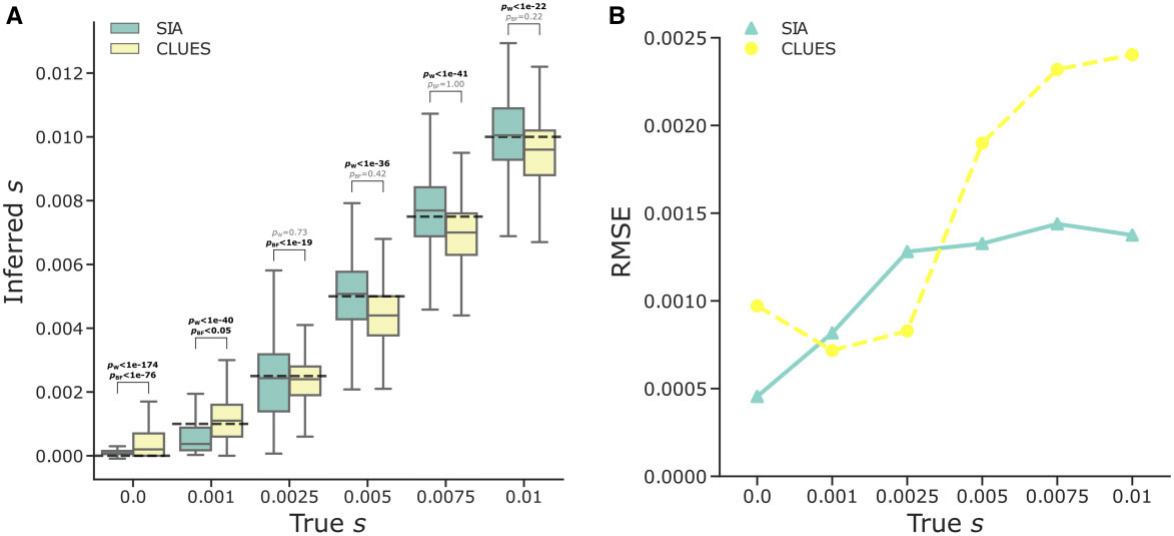
Predictions of selection coefficients on simulated regions using SIA and CLUES based on true genealogies. (*A*) The distribution of inferred selection coefficients for each method under each model condition are reported using a box plot. The box plot for each method reports these five statistics (from bottom to top): minimum, first quartile, median, third quartile, and maximum. The *y*-axis shows the inferred selection coefficient, whereas the *x*-axis shows the true selection coefficient. The dashed-black line indicates the true selection coefficient for each model condition. The simulations are based on the CEU demographic model and true genealogies were used as input to both methods. Each model condition (i.e., box plot) represents a set of 400 independent simulations. The mean ranks and variances of the distributions of inferred *s* were compared using the Wilcoxon signed-rank test (*p*_W_) and the Brown–Forsythe test (*p*_BF_), respectively. (*B*) The root mean square error (RMSE) for each method under each model condition evaluated on 400 independent simulations.

We began by supplying both methods with true genealogies, in order to later disentangle the error deriving from the ARG inference step from other sources of error (see Discussion). We found that SIA identified regions under neutrality with approximately no bias (median inferred *s* = 7.5e−05; fig. 3). Similarly, SIA correctly inferred the selection coefficient for regions under moderate to strong selection (*s*∈ {0.0025, 0.005, 0.0075, 0.01}) with the median inferred *s* deviated from the true *s* by at most 3%. On the other hand, SIA somewhat underestimated the selection coefficient (median inferred *s* = 0.00037) for the weak selection regime (true *s* = 0.001), likely owing to limits in the training set within that selection regime (see Discussion). We further binned the results by segregating frequency and selection coefficient and found that, in general, the variance in estimates of *s* for SIA (as well as CLUES) tended to decrease as the segregating frequency of the beneficial allele increased (supplementary fig. S3, Supplementary Material online).

CLUES performed roughly similarly to SIA in this experiment, but tended to slightly overestimate *s* for the neutral regions (i.e., true *s* = 0) and underestimate *s* for the moderate to high selection regimes (i.e., true *s* = 0.005, 0.0075, and 0.01). Under these conditions, SIA’s median predictions of *s* were noticeably closer to the true values (fig. 3*A*). At the same time, CLUES performed slightly better than SIA in weak selection regimes (i.e., true *s* = 0.001 and 0.0025) (fig. 3). Overall, SIA (RMSE = 9.52e−4) achieved a lower error in estimating *s* than CLUES (RMSE = 1.44e−3), when true genealogies were used as input to both methods (Wilcoxon signed-rank test for difference in mean of squared error, *P* = 1.25e−42). This finding potentially reflects the benefit of linkage information utilized by SIA through the additional flanking genealogies (see Discussion).

### Selection Coefficient Inference Using Inferred Gene Trees

To account for gene-tree uncertainty, we next used ARGs inferred with Relate, which is scalable to the size of the training data set for SIA (see Materials and Methods), as input to SIA and CLUES and compared their performance on CEU simulations. Using a reduced sample size of 32 haplotypes, we additionally compared SIA with CLUES supplied with genealogies sampled using ARGweaver. Furthermore, we compared both methods with a supervised ML method, ImaGene (see supplementary fig. S23, Supplementary Material online), that operates directly on an image of the alignment itself. ImaGene does not require gene trees as input and instead uses a convolutional neural network (CNN) to perform dimensionality reduction of the sequence alignment, allowing for accurate and efficient classification and regression.

**Fig. 4.**
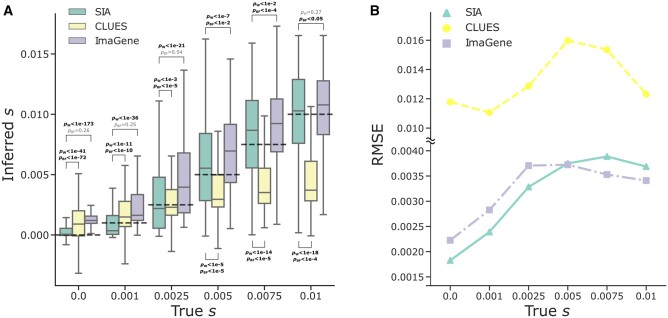
Predictions of selection coefficient on simulated regions using SIA and CLUES based on inferred genealogies, and ImaGene. (*A*) The distribution of inferred selection coefficients and (*B*) root mean square error (RMSE) for each method under each model condition. The simulations are based on the CEU demographic model where inferred genealogies were used as input to SIA and CLUES, whereas sequence alignments were used as input to ImaGene. Figure layout and description are otherwise similar to figure 3.

Overall, we found that SIA and ImaGene outperformed CLUES in these experiments (fig. 4). CLUES tended to underestimate selection coefficients for the moderate-to-strong selection regimes, to a greater extent compared with the case where true genealogies were used for inference (figs. 3*A* and 4*A*). This decrease in performance of CLUES evidently derives from error at the ARG reconstruction step. SIA, on the other hand, appeared to be more robust to the same ARG reconstruction error, and maintained an advantage even when CLUES was provided posterior samples of genealogies from ARGweaver (supplementary fig. S5, Supplementary Material online). ImaGene performed remarkably similarly to SIA, given that it relies solely on the sequence alignment. SIA exhibited lower error at neutral sites and sites with low-to-moderate values of *s*, whereas ImaGene prevailed at sites under strong selection (fig. 4*B*). Nevertheless, SIA showed a slightly smaller overall RMSE (2.75e−3) compared with ImaGene (2.91e−3) (Wilcoxon signed-rank test, *P* = 6.18e−38), and in particular, SIA produces estimates of *s* much closer to 0 for neutral loci. Notably, in this case both SIA and ImaGene were trained with simulations under the same uniform distribution of *s* values (see Materials and Methods). A different choice of training distribution could impact their performance across selection regimes (see Discussion). Furthermore, we binned the results of these methods by both the segregating frequency and the selection coefficient (see supplementary fig. S4, Supplementary Material online) and again found that in general they exhibit higher variance under low segregating frequency of the beneficial allele. As before, we also tested our regression framework on true and inferred gene trees of test sets simulated under the *Sporophilahypoxantha* demographic model (see supplementary fig. S6, Supplementary Material online). We found that SIA was approximately unbiased for the moderate (*s* = 0.005) and high (*s* = 0.01) selection regimes but appeared to overestimate the selection coefficient for regions under weak selection (*s* = 0.001 and 0.0025), when both true and inferred genealogies were used as input. Furthermore, SIA appeared to overestimate the selection coefficient for neutral regions when inferred gene trees were used as input, whereas it was approximately unbiased for true gene trees.

### Performance on Selection Coefficient Prediction with Different Sample Sizes

To explore the tradeoffs associated with the use of larger data sets, we examined the performance of SIA under different sample sizes, assuming a constant-sized demographic model (*N*_e_ = 10,000). Supplementary figure S7, Supplementary Material online shows the error in selection coefficient inference on a held-out test set, stratified by the age of the allele (supplementary fig. S7*A* and *B*, Supplementary Material online) and present-day derived AF (supplementary fig. S7*C* and *D*, Supplementary Material online) at the site of interest. We observed that sites with low frequency (AF < 0.33) and more recent (onset < 0.2 × 2 *N*_e_ generations) alleles experience the most significant reduction in error as sample size increases. Notably, the performance of SIA on more ancient alleles (onset > 0.2 × 2 *N*_e_ generations) had little to no improvement as the sample size increased from 32 to 254. These observations are in line with the expectation that having more samples improves the chance of capturing low-frequency alleles, but provides limited information about more ancient events. The reason for this age-dependency is that, looking backwards in time, most lineages coalesce rapidly and only a few survive to more ancient epochs, in a manner that depends only weakly on the sample size. It may be useful to consider these observations when choosing the sample size for use in studying selection in a particular context (see Discussion).

### Inference of AF Trajectory

We further adapted the deep-learning architecture of SIA to model the AF trajectory at a site by retaining the output of the LSTM at each time point (supplementary fig. S1, Supplementary Material online; see Materials and Methods). We then evaluated the performance of SIA in the inference of the AF trajectory using simulations under the CEU demography across a range of selection coefficients and current DAFs. SIA was largely able to capture the expected trend of more rapidly increasing AF under stronger selection (supplementary figs. S8 and S11, Supplementary Material online). In addition, AF estimates by SIA using both true and inferred genealogies were generally unbiased, although AF at more recent time points tended to be slightly underestimated when data was simulated under weaker selection. AF estimates also appeared to be more accurate in terms of variance for alleles under stronger selection (supplementary figs. S9 and S12, Supplementary Material online). As expected, the variance of AF estimates tended to increase going further back in time (supplementary figs. S9 and S12, Supplementary Material online). We also observed that overall SIA tended to produce more accurate AF estimates than CLUES (supplementary figs. S9 and S10, Supplementary Material online).

### Model Performance on Simulations with Mis-specified Demographic Models

To evaluate the robustness of SIA to mismatches between the demographic parameters used for simulating training data and the true underlying demography of real data, we tested the method on the selection-coefficient inference task with data sets simulated under a range of alternative parameters. Each aspect of this model mis-specification was assessed independently of the others. In particular, the mis-specified data sets contained simulations under 1) combinations of population mutation (*θ*) and recombination (*ρ*) rates sampled beyond the range used for the training data (supplementary figs. S13 and S16, Supplementary Material online); 2) various alternative demographic scenarios (supplementary figs. S14, S17, and S19, Supplementary Material online); and 3) various effective population sizes (supplementary figs. S15 and S18, Supplementary Material online). We compared the performance of SIA on these mis-specified data sets with that of CLUES (Stern et al. 2019), supplying both methods with the true genealogies. We consider CLUES the “silver standard” when it comes to robustness because it is unsupervised and therefore should not be susceptible to mis-specified training data compared with supervised learning methods such as SIA. Overall, we found that both CLUES and SIA were reasonably robust to model mis-specification (supplementary figs. S13–S15, Supplementary Material online), although the performance of both methods inevitably declined when tested on severely mis-specified data (supplementary fig. S15, Supplementary Material online). Interestingly, SIA tended to overestimate selection coefficient when the true *N*_e_ was much smaller than that used for training, and underestimate it when the true *N*_e_ was much larger, whereas CLUES did the opposite (supplementary fig. S15, Supplementary Material online). Because the CLUES likelihood model of AF transition is parameterized by the population-scaled selection coefficient (*α* = 2*Ns*), a larger *N*_e_ likely appears to CLUES as equivalent to a higher *s*. On the other hand, features used by SIA capture broad information of coalescence and linkage in the ARG, and therefore can be distorted by mis-specified *N*_e_ in more subtle ways (see Discussion). Using the same mis-specified data set, we also ran SIA with Relate-inferred genealogies and compared its performance with that of the genotyped-based deep-learning model ImaGene (Flagel et al. 2019; Torada et al. 2019). In general, SIA appeared to be more robust to model mis-specifications, achieving an overall RMSE of 0.00362, 0.00318, and 0.00374 in the mis-specified *θ*/*ρ*, demography, and *N*_e_ experiments, respectively, compared with ImaGene, whose RMSE was 0.00416, 0.00330, and 0.00462 in the corresponding experiments (supplementary figs. S16–S18, Supplementary Material online). The advantage of SIA was particularly noticeable in cases of mis-specified demographic parameters (supplementary figs. S17 and S18, Supplementary Material online). Notably, SIA exhibited reduced bias when working with inferred genealogies compared with true genealogies, under conditions of extremely mismatched *N*_e_ (compare supplementary figs. S15 and S18, Supplementary Material online).

### Model Prediction at Genomic Loci of Interest in CEU Population

We then applied the SIA model to identify selective sweeps and infer selection coefficients at selected genomic loci in the 1000 Genomes CEU population. These loci included the canonical example of selection at the *MCM6* gene, which regulates the neighboring *LCT* gene and contributes to the lactase persistence trait (Bersaglieri et al. 2004), the *ABCC11* gene regulating earwax production, several pigmentation-related genes, as well as genes associated with obesity, diabetes and addiction (table 1).

**Table 1.** List of Genomic Loci of Interest Along with Their Derived Allele Frequencies, Sweep Probabilities, and Selection Coefficients Inferred by SIA in the 1000 Genomes CEU Population.

Gene	SNP ID	Chr	**Position** ^a^	Derived *f*	*P* (sweep)	Selection Coefficient (95% CI)
*LCT* (Bersaglieri et al. 2004)	rs4988235	2	136608646	0.74	0.999	[0.01019, 0.01056]
*OCA2* (Han et al. 2008; Sturm et al. 2008)	rs12913832	15	28365618	0.77	0.750	[0.00539, 0.00575]
*MC1R* (Sulem et al. 2007; Han et al. 2008)	rs1805007	16	89986117	0.12	0.949	[0.00362, 0.00384]
*ABCC11* (Yoshiura et al. 2006)	rs17822931	16	48258198	0.13	0.620	[0.00034, 0.00036]
*ASIP* (Eriksson et al. 2010)	rs619865	20	33867697	0.12	0.777	[0.00172, 0.00197]
*TYR* (Sulem et al. 2007; Eriksson et al. 2010)	rs1393350	11	89011046	0.24	0.616	[0.00085, 0.00135]
*KITLG* (Sulem et al. 2007)	rs12821256	12	89328335	0.13	0.869	[0.00183, 0.002]
*TYRP1* (Kenny et al. 2012)	rs13289810	9	12396731	0.37	0.144	[0.00004, 0.00006]
*TTC3* (Liu et al. 2010)	rs1003719	21	38491095	0.62	0.011	[0, 0]
*OCA2*	rs7495174	15	28344238	0.94	0.013	[0, 0.00005]
*TCF7L2* (Lyssenko et al. 2007)	rs7903146	10	114758349	0.69	0.035	[0, 0]
*ANKK1* (Spellicy et al. 2014)	rs1800497	11	113270828	0.80	0.045	[0, 0]
*FTO* (Frayling et al. 2007)	rs9939609	16	53820527	0.56	0.011	[0, 0]

a Genomic coordinates in GRCh37 (hg19) assembly.

**Fig. 5.**
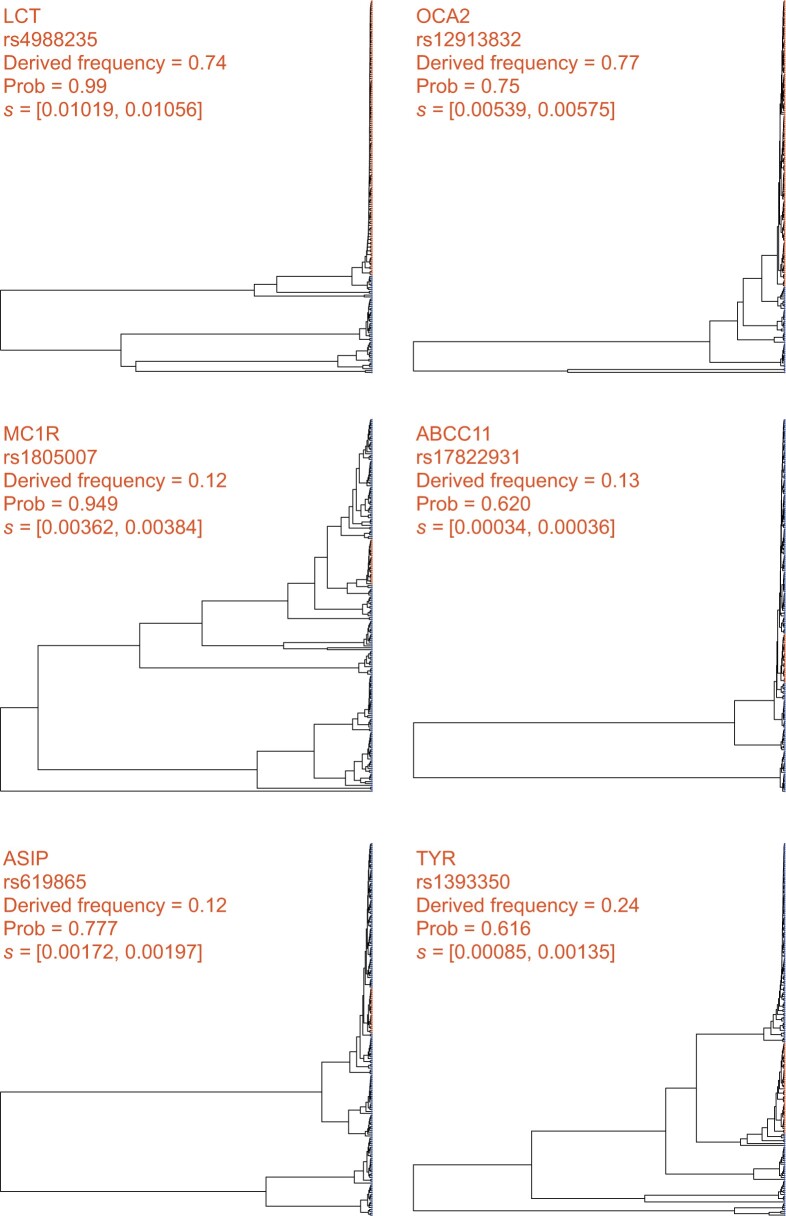
Local genealogies at six loci inferred to be under positive selection in the 1000 Genomes CEU population. Gene name, RefSNP number, derived AF, SIA-inferred sweep probability and SIA-inferred selection coefficient range for each locus are indicated at the top of each panel (see table 1 for more details). Taxa carrying the ancestral and derived alleles are colored in blue and orange, respectively.

For *LCT*, SIA detected a strong signal of selection at the nearby SNP that has been associated with the lactase persistence trait (rs4988235). At this SNP, SIA inferred a sweep probability close to 1 and a selection coefficient >0.01, making this one of the strongest signals of selection in the human genome. A close examination of the local genealogy at this site reveals a clear pattern indicative of a selective sweep––a burst of recent coalescence among the derived lineages (orange taxa are the lineages carrying the derived allele) is clearly visible from the tree (fig. 5).

At a number of pigmentation genes (Sulem et al. 2007; Han et al. 2008; Sturm et al. 2008; Liu et al. 2010; Kenny et al. 2012), SIA detected signals of moderate selection, including *MC1R* (rs1805007, P(sweep) = 0.95, s ≈ 0.0037), *KITLG* (rs12821256, P(sweep) = 0.87, s ≈ 0.0019), *ASIP* (rs619865, P(sweep) = 0.78, s ≈ 0.0019), *OCA2* (rs12913832, P(sweep) = 0.75, s ≈ 0.0056), and *TYR* (rs1393350, P(sweep) = 0.62, s ≈ 0.0011). In addition, SIA identified a weak signal of selection at a SNP in the *ABCC11* gene (rs17822931), which influences earwax and sweat production (Yoshiura et al. 2006), with a selection coefficient of around 0.00035. There are few other estimates for these genes available for comparison, but, notably, our estimate for *LCT* of *s* ≈ 0.01 is consistent with a previous estimate on the order of 0.01–0.1 (Bersaglieri et al. 2004), and with recent studies of ancient DNA samples (Mathieson and Mathieson 2018; Mathieson 2020) suggesting a value closer to 0.01. Our estimates suggest that selection at the pigmentation loci is considerably weaker than at *LCT*, in contrast to previous estimates for these loci, which covered a wide range but were generally considerably larger (ranging from 0.02 to 0.1) (Wilde et al. 2014). Interestingly, CLUES estimated *s* at the *OCA2* locus to be on the order of 0.001 (roughly similar to SIA’s estimate of 0.0056), but *s* at the *KITLG*, *ASIP*, *TYR* loci to be >0.01 (in comparison to SIA’s considerably smaller estimates of 0.0019, 0.0019, and 0.0011) (Stern et al. 2019). The apparent discrepancy between the estimates may be partially due to the fact that the two methods used samples from two different populations (CEU for SIA and GBR/British for CLUES).

**Fig. 6.**
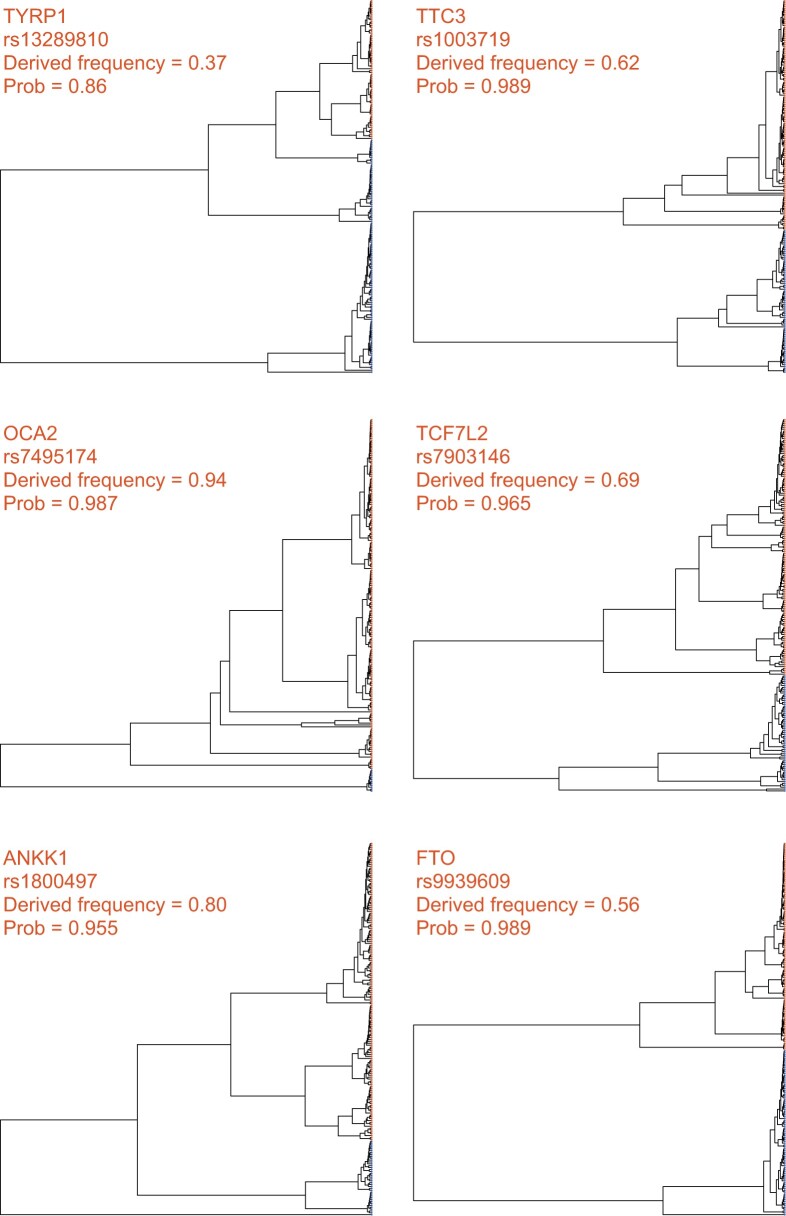
Local genealogies at six loci lacking signal of positive selection in the 1000 Genomes CEU population. Gene name, RefSNP number, derived AF and probability of neutrality inferred by SIA for each locus are indicated at the top of each panel (see table 1 for more details). Taxa carrying the ancestral and derived alleles are colored in blue and orange, respectively.

On the other hand, SIA did not detect significant evidence of positive selection at several disease-associated loci (rs7903146/*TCF7L2*, rs1800497/*ANKK1*, and rs9939609/*FTO*) or at several other pigmentation loci (rs13289810/*TYRP1*, rs1003719/*TTC3*, and rs7495174/*OCA2*) (table 1). Notably, allele frequencies at these six loci tend to be similar in African and European populations (Marcus and Novembre 2017), suggesting that they are not likely to be under strong environment-dependent positive selection, although it is possible that they have experienced very recent selective pressure that SIA lacks the power to detect (see Discussion). Notably, *TYRP1* and *TTC3* also lacked signals of selection in the CLUES analysis. Compared with the genealogies at sweep sites (fig. 5), the trees at these putatively neutral loci lack the distinctive signature of recent bursts of coalescence among derived lineages (fig. 6).

### Southern Capuchino Species Analysis

**Fig. 7.**
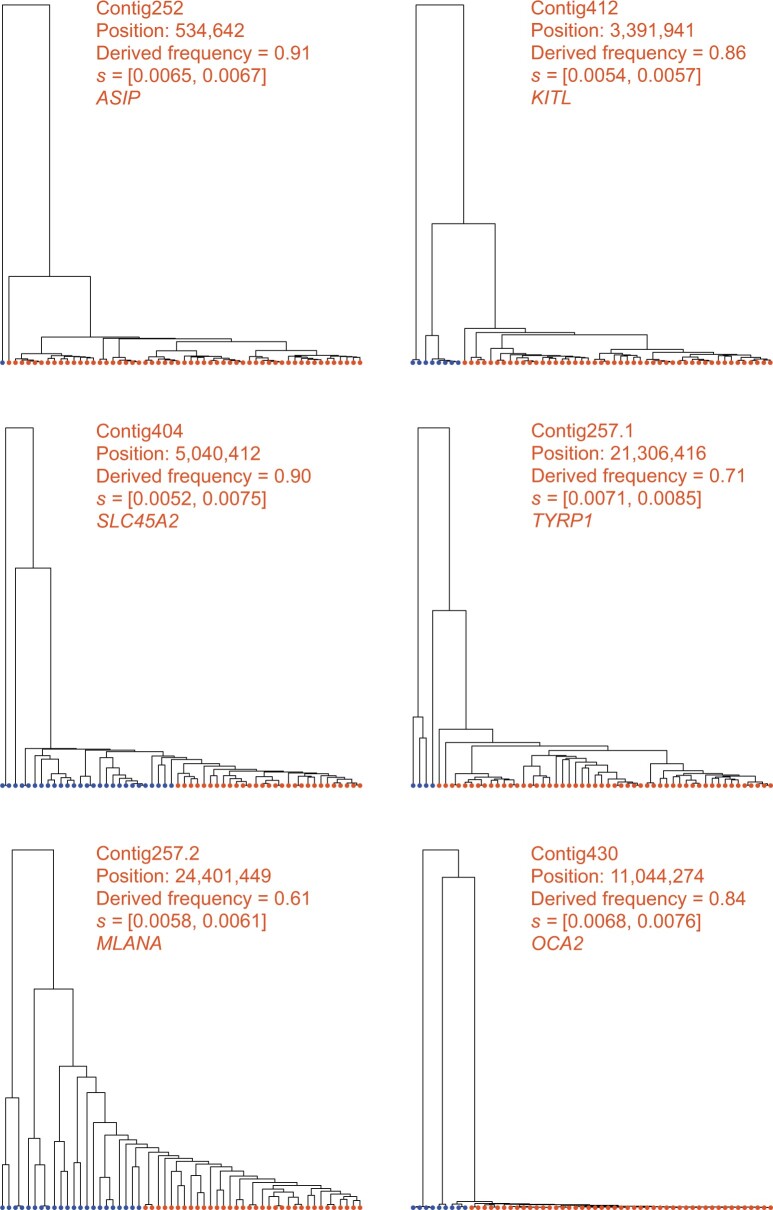
Local genealogies at six loci inferred to be under positive selection in *S. hypoxantha*. Contig name, position of SNP, derived AF, SIA-inferred selection coefficient range, and the pigmentation gene closest to the locus in question are indicated at the top of each panel. Haploid genomes carrying the ancestral and derived alleles are colored in blue and orange, respectively.

Our previous study of southern capuchino seedeaters made use of the full ARG and ML to detect and characterize selective sweeps, and suggested that soft sweeps are the dominant mode of adaptation in these species (see Materials and Methods for more details). To further characterize the targets and strengths of positive selection in these species, we applied SIA to polymorphism data (Turbek et al. 2021) for *S. hypoxantha*, and adopted a conservative approach by reporting only sites with DAF ≥ 0.5, SIA-inferred *s* ≥ 0.0025, and SIA-inferred sweep probability ≥0.99 (see Materials and Methods). In addition to loci near top *F*_ST_ peaks and known pigmentation-related genes (table 2), we identified many more sites under positive selection located outside the previously scanned *F*_ST_ peaks, amounting to a total of 15,551 putative partial soft sweep sites across the 333 scanned scaffolds for *S. hypoxantha*. These sites can be prioritized for further evaluation and downstream analysis. Notably, SIA enabled us to distinguish between selection at regulatory and coding sequences, and we found that sweep loci near *F*_ST_ peaks and pigmentation genes fall mostly in noncoding regions (table 2). We additionally surveyed all putative sweep sites identified by SIA and found that they are indeed enriched in noncoding regions (Fisher’s exact test, *P* = 6.80 × 10^−5^), particularly noticeable in the “near-coding” regions (supplementary fig. S22, Supplementary Material online). Consistent with the observation that the most highly differentiated SNPs among taxa are noncoding (Campagna et al. 2017; Turbek et al. 2021), our finding suggests that positive selection may act on *cis*-regulatory regions to drive differentiation and the subsequent speciation process. Furthermore, we examined many individual predictions in detail, considering the local trees inferred by Relate at these high-confidence predictions (fig. 7). We found, in numerous cases, that these sweeps had distinct genealogical features, displaying evidence of a burst of coalescence events, corresponding to unusually large and young clades. Prominent examples include predictions near pigmentation-related genes *ASIP*, *KITL*, *SLC45A2*, and *TYRP1*.

**Table 2.** The Top 25 *F*_ST_ Peaks Identified in Hejase, Salman-Minkov, et al. (2020) Along with the Number of Partial Soft Sites in *S. hypoxantha* Identified for Each Scaffold Using SIA.

Scaffold	Start Position (Mb)	End Position (Mb)	Length (kb)	No. of Partial Soft Sites^a^
59	5.74	5.86	120	11
118	7.16	7.22	60	5
252	0.40	0.54	140	3
257.1	21.24	21.78	540	26
257.2	24.40	24.84	440	43
257.3	28.66	28.96	300	10
257.4	31.30	31.38	80	8
257.5	5.78	6.20	420	25 (1)
263	0.00	0.58	580	31
308	0.04	0.20	160	0
404.1	5.04	5.84	800	115 (7)
404.2	10.76	10.96	200	30
412	3.38	3.62	240	15
430	10.98	11.10	120	24
567	2.50	2.80	300	0
637.1	6.00	6.32	320	2
637.2	6.84	6.92	80	4
762	1.65	1.73	80	30
766	1.98	2.10	120	1
791	9.90	9.98	80	15
1,717	0.92	0.98	60	7
3,622	0.96	1.36	400	8
1,635	3.71	3.75	40	4
1,954	2.8	2.9	100	17
579	0.1	0.16	60	0

Note.—To avoid cases with limited power, we focused on sites with segregating frequency ≥ 0.5, SIA-inferred *s* > 0.0025, and SIA-inferred sweep probability ≥ 0.99.

a The number of sweep sites in coding regions is shown in parenthesis.

## Discussion

The ARG is useful for addressing a wide variety of biological questions ranging from inferring demographic parameters to estimating allele ages. SIA exploits the particular utility of the ARG for accurate inference of positive selection in a way that makes use of the full data set, as opposed to traditional summary statistics, which necessarily discard substantial information. Direct use of the ARG improves upon traditional summary statistics in two key ways. First, it enables consideration of the temporal distribution of coalescence and recombination events in the history of the analyzed sequences, in contrast to traditional summary statistics that simply average over these coalescence and/or recombination events. In addition, ARG-based methods provide better spatial resolution by separately examining individual genealogies and the recombination breakpoints between them, rather than averaging across windows containing unknown numbers of genealogies. These detailed patterns of coalescences and linkage enable the ARG-based approaches to capture a more localized and fine-grained picture of selection (e.g., infer selection coefficient and AF trajectory) as well as to achieve a better classification performance. This performance advantage is particularly noticeable at lower DAFs and when selection is weak, a regime where previous methods for selection inference fall short (fig. 2).

At the same time, the supervised ML approach sets SIA apart from another ARG-based method, CLUES, which approximates a full likelihood function for ARGs in the presence of selection using importance sampling and an HMM. Although the accuracy of both SIA and CLUES degraded when using inferred genealogies compared with true genealogies, reflecting the error and uncertainty at the ARG inference step, SIA appeared to be more robust to gene tree uncertainty (figs. 3 and 4). One possible reason for this observation is that CLUES effectively assumes that the selection coefficient at the focal site is conditionally independent of the flanking trees given the focal tree. This assumption should hold in the presence of fully specified genealogies, but it may make CLUES more sensitive to errors in the inferred genealogies. In other words, through its use of supervised learning, SIA may be able to compensate for the effects of genealogy inference error on its estimation of the selection coefficient by also directly considering the flanking trees and LD-related patterns among them. Still, the drop in accuracy observed across methods underscores the dependency of ARG-based approaches on the ARG inference method. For this reason, we anticipate that SIA may benefit substantially from further improvement in ARG inference tools (see Hejase, Dukler, et al. [2020]).

The ARG-based feature set distinguishes SIA from other supervised ML approaches for characterizing selective sweeps. SIA uses local topological features of the ARG, which are more informative than the SFS- or LD-based summary statistics employed by ML methods such as S/HIC, SFselect, and evolBoosting. Using simulations, we demonstrated that the SIA classifier outperformed a deep-learning method that aggregates these traditional summary statistics (fig. 2). We also compared SIA with ImaGene, which represents another flavor of supervised learning methods, inspired by the recent rise of CNNs for image recognition. ImaGene encodes sequence alignments as images for powerful population genetic inferences with CNNs and provides a state-of-the-art benchmark to compare against. We found that ImaGene performs remarkably well across a wide range of simulations, but SIA does appear to be somewhat less biased and more robust to model mis-specification than ImaGene. The evolutionary information in the ARG is implicit in the sequence alignment but some of this information may be difficult for a brute-force ML model to discover directly.

We demonstrated that utilizing the ARG granted SIA considerably improved performance over deep-learning models solely employing traditional summary statistics. However, a possible drawback of an ARG-based model is the potentially prohibitive computational overhead incurred by ARG inference, especially as sample size grows. Picking a sample size when running SIA involves a tradeoff between scalability (fewer samples, faster ARG inference) and performance (more samples, slower ARG inference). We have found that SIA can infer selection coefficients reasonably well with as few as 16 haplotypes. Including more samples did improve performance but with a sublinear reduction in error (supplementary fig. S7, Supplementary Material online). Therefore, a sample size from a few dozen to a few hundreds—well within the capabilities of most modern ARG inference methods—strikes a good balance between performance and scalability. Moreover, we found that larger sample sizes improved prediction performance primarily for alleles at lower frequencies but had little impact on the performance for more ancient alleles (as most lineages would have already coalesced going further back in time) (supplementary fig. S7, Supplementary Material online). This observation suggests that the choice of the sample size when applying SIA should be guided by the biological question of interest––ancient selection can be studied with just a handful of samples, whereas a larger sample size is better suited to detect more recent sweeps. Notably, the addition of ancient DNA samples could potentially enable selection to be inferred over much longer time scales. It should be possible to accommodate them with a relatively straightforward extension of the method.

Like other supervised learning methods, SIA relies on simulations to generate training data. In order to apply SIA in a particular population, a fresh set of training data tailored to that population needs to be simulated. Although it takes on the order of 100 CPU hours to simulate the training data compared with ten CPU hours to train the model (see Materials and Methods), simulations can be easily distributed across multiple machines as each of them runs independently. Another potential drawback common to supervised methods is that they could be biased by subjective choices of simulation parameters. For example, SIA and ImaGene cannot make accurate predictions of selection coefficients outside the range represented in the training data (supplementary fig. S20, Supplementary Material online), whereas unsupervised methods such as CLUES are not limited to a predefined range (supplementary fig. S21, Supplementary Material online). This problem could be circumvented by training on an extended range of *s*. Similarly, the tendency of SIA to underestimate the selection coefficient for sites under weak selection (figs. 3 and 4) could be mitigated by augmenting the training set with simulations densely sampled from the weak selection regime. A more subtle issue, however, arises when the underlying generative process of the real data does not match the assumptions made for the simulations of the training data, potentially compromising the accuracy of the method when applied to real data. Thus, we tested SIA on simulations with parameters mismatching those used in the training procedure. In general, we found that SIA was fairly robust to alternative parameter values, although, as expected, performance did degrade somewhat under severely mis-specified models. Notably, SIA achieved a similar level of robustness to model parameter mis-specification as the unsupervised (i.e., not relying on training data) likelihood method CLUES, yet outperformed the supervised deep-learning method ImaGene.

Applying SIA to the CEU panel from the 1000 Genomes Project yielded several noteworthy findings at loci with known ties to phenotypes of interest. In addition to confirming the canonical signal of selective sweep at the *LCT* locus, SIA detected a novel signal of selection at a GWAS SNP in the *MC1R* gene associated with red hair color, contrasting a previous study that could not find evidence of selection at *MC1R* in the European population (Harding et al. 2000). The derived allele at this locus segregates at around 10% in the CEU population but is nearly absent in non-European populations (Marcus and Novembre 2017). In addition, at the *MC1R* locus the Relate test statistic for selection (Speidel et al. 2019), which tends to perform particularly well at low segregating frequencies (fig. 2), falls slightly below the significance threshold of 0.05, supporting the evidence of positive selection at this locus. SIA also detected evidence of selection at a SNP in the *ABCC11* gene reported to be the determinant of wet versus dry earwax as well as sweat production, mirroring the signal of selection previously found in the East Asian population (Ohashi et al. 2011), although selection in the CEU population appeared to be much weaker. In addition, SIA identified selection at a few other pigmentation-related loci, yet determined previously identified SNPs in the *TYRP1* and *TTC3* genes to be largely free from selection (table 1). These results were consistent with a previous study (Stern et al. 2019), which reported similar results for these pigmentation-related loci, albeit in a slightly different population (GBR). SIA notably did not detect positive selection at GWAS loci in the *TCF7L2* gene associated with type-2 diabetes, the *ANKK1* gene implicated in addictive behaviors, and the *FTO* gene associated with obesity. Overall, this empirical study with the 1000 Genomes CEU population has illustrated how SIA can be applied to assess natural selection at the resolution of individual sites, suggesting that it may be useful in prioritizing GWAS variants for further scrutiny.

In our previous work on southern capuchino seedeaters (Hejase, Salman-Minkov, et al. 2020) (see Materials and Methods), we applied newly developed statistical methods for ARG inference and ML for the prediction of selective sweeps. We found evidence suggesting that a substantial fraction of soft sweeps is partial but had limited power to identify them (i.e., average accuracy of 56%). SIA considerably improved our characterization of positive selection in the southern capuchino species in two key ways. The SIA framework performs inference of selection directly from genealogies instead of traditional summary statistics, and in doing so achieved an accuracy of up to 96% in detecting partial soft sweeps. Consequently, we found abundant evidence of soft sweeps beyond the previously scanned *F*_ST_ peaks, and additionally were able to estimate their selection coefficients. Importantly, SIA also took the analysis of selection beyond broad genomic windows containing sweeps to the identification of specific putative causal variants. We took advantage of this substantial improvement in genomic resolution and analyzed the distribution of these sweep sites, which revealed that positive selection on regions that likely contain *cis*-regulatory elements plays a role in driving the differentiation and speciation of southern capuchino seedeaters.

Although we believe SIA represents an important step forward in the use of the ARG for ML-based selection inference, there remain several possible avenues for improvement. For example, SIA currently uses a point-estimate of the ARG, rather than a distribution, and therefore does not explicitly take gene-tree uncertainty into account. Instead, the uncertainty of the inferred parameters is estimated with neural network dropouts (Gal and Ghahramani 2016). The variance of parameter inference could alternatively be assessed from uncertainty in genealogy reconstruction by resampling coalescent times with Relate (Speidel et al. 2019), and moreover resampling trees from the posterior distribution of ARGs with ARGweaver (Rasmussen et al. 2014). Thus, it may be enlightening to compare these different approaches to analyzing uncertainty. Likewise, SIA will greatly benefit from better algorithms for ARG reconstruction that balance accuracy with scalability and can handle thousands of genomes. In addition, the SIA framework was applied in the context of single-locus selective sweeps, but could be extended to study polygenic selection, by making use of summary statistics from genome-wide association studies (as in Stern et al. [2021]) and adapting the architecture of our neural network to account for selection acting at multiple sites. Finally, the robustness of SIA to model mis-specifications can be further improved by ensuring the simulated data is generated under a distribution that is compatible with the real target data set. We anticipate that the continual advancement in ARG inference methods has the potential to open up many new applications for this flexible and powerful model of ARG-based deep learning in population genetics.

## Materials and Methods

### Simulated Data Sets Used for Training and Testing the SIA Model

Training and testing data sets were generated using discoal (Kern and Schrider 2016) by simulating 1,000,000 regions of length 100 kb for each model we considered (i.e., “neutral” or “hard sweep”). Aside from these regions, 2,000 were simulated for validation and 5,000 were simulated for testing. The number of sampled sequences was selected to match the number of individuals in the CEU population in the 1000 Genomes data set. Thus, a total of 198 haploid sequences were sampled. Simulations used a demographic model based on European demography (Tennessen et al. 2012). In non-neutral simulations, selection was applied to a single focal site located in the middle of the simulated region. We sampled each of the main demographic and selection parameters from a uniform distribution: 1) mutation rate *μ* ∼ *U*(1.25e−08, 2.5e−08); 2) recombination rate *ρ* ∼ *U*(1.25e−08, 2.5e−08); 3) selection coefficient *s* ∼ *U*(0.0001, 0.02); and 4) segregating frequency of the site under selection *f* ∼ *U*(0.01, 0.99). The total storage footprint for the simulations was 1.6TB. The average cost of one simulation was 0.53 s, amounting to a total of 148 CPU hours to simulate the entire training set. The cost of simulation was mitigated by parallelization across multiple compute nodes.

### ARG Feature Extraction

For each target variant, we extracted the corresponding gene tree from the ARG, then overlaid it with 100 discrete timepoints. These timepoints were fixed across all trees in an approximately log-uniform manner that resulted in finer discretization of more recent time scales (as in Rasmussen et al. [2014]). We considered biallelic sites only and assumed no recurrent mutations; thus, each mutation was assumed to occur on the branch of the tree where the ancestral allele switches to the derived. For each timepoint, we calculated the number of active ancestral and derived lineages. Furthermore, we computed the number of all active lineages (not distinguishing between ancestral and derived) at the same set of predefined timepoints in the two left- and right-flanking gene trees to account for linkage disequilibrium. We experimented with alternative numbers of flanking gene trees and found that the SIA model with two flanking gene trees (RMSE = 0.0027) outperforms a model with one (RMSE = 0.0029) or no (RMSE = 0.0030) flanking gene tree. Generally, more gene trees provide SIA with richer linkage information and thus improve its ability to estimate the effect of positive selection on a locus. The exact threshold of diminishing returns, however, can be computationally costly to establish. We therefore opted to include two flanking gene trees while noting that the user can control this hyperparameter when running SIA.

In the end, the ARG feature for each locus consisted of a 600-dimensional vector, which was then used as input to an RNN. The features for each simulated sweep region were extracted from the sweep site (by default at the center in all simulations) whereas the features for a simulated neutral region were extracted from a variant site (randomly chosen) with a predefined matched derived AF. The features for each genomic locus of interest in the CEU population were extracted from all variant sites at that locus having a derived AF of >0.05.

### Training an RNN to Predict Different Modes of Selection

An RNN was applied to the simulated training data sets to learn a classification or regression model for the task at hand. We used a LSTM, a particular form of RNN, to accommodate the temporal nature of our features, account for long-term dependencies, and tackle the vanishing gradient problem observed in traditional RNNs. Our model had 100 timepoints with the final target output depending on the use of classification or regression. For the classification task, the final target output is a binary class label predicting whether a region is under selection or neutrality. For the regression task, the final target output is a continuous value, representing the selection coefficient or the time of selection onset. We also took a many-to-many approach to model the AF trajectory for the site under selection. The *Keras* software was used to train and test the model. We used a two-stacked LSTM to account for greater model complexity where the number of units in each stack was set to 100 and the hyperbolic tangent (*tanh*) was used as an activation function. The *Adam* optimization method with its default operating parameters was used to update the network weights. For the classification task, the *Softmax* activation function was applied on the final dense layer and the *binary_crossentropy* was used to compute the cross-entropy loss between true labels and predicted labels. For the regression task, the *linear* activation function was applied on the final dense layer and the *mean_squared_error* loss was used. The SIA deep-learning model took on average 7–10 h to train on a single GPU node with 32 GB memory and four threads, whereas applying the trained model for prediction took less than a minute.

### Estimation of Confidence Intervals

To turn our single-valued regression model into one capable of returning a distribution of predictions of *s*, we reused the dropout technique that is typically used during training. Dropout enables a fraction of nodes to be randomly “turned off” in a certain layer, which assists in the regularization of the model and helps prevent overfitting. We applied dropout during inference, enabling us to sample a “thinned” network to generate a sample prediction. By repeatedly sampling thinned networks, we generated a distribution of predictions and then computed confidence intervals based on this distribution (Gal and Ghahramani 2016).

### ARG Inference

Relate (Speidel et al. 2019) (v1.0.17) was used for inferring ARGs underlying simulated genomic samples as well as the CEU population in the 1000 Genomes data set. For simulations under the Tennessen *et al.* demography (Tennessen et al. 2012), Relate was run with the true simulation parameters (*μ*, *ρ*, and *N_e_*) specified; whereas for genomic loci of the CEU population, Relate was run with a mutation rate of 2.5 × 10^−8/^base/generation (−m 2.5e−8), a constant recombination map of 1.25 × 10^−8^/base/generation and a diploid effective population size of 188,088 (−N 376176). The choice of mutation rate follows Stern et al. (2019) based on estimates from Nachman and Crowell (2000). Although some more recent estimates have been lower (Scally and Durbin 2012), these differences in mutation rate are unlikely to have a major effect on our selection inference because SIA appears to be fairly robust to mis-specification of mutation rate (supplementary figs. S13 and S16, Supplementary Material online). For simulations and genomic loci of the *S. hypoxantha* population, Relate was run with *μ* = *ρ* = 1 × 10^−9^/base/generation and a diploid *N_e_* of 130,000. The branch lengths of Relate-inferred genealogies were estimated iteratively with the “*EstimatePopulationSize.sh*” script in the Relate package. Specifically, population size history was inferred from the ARG, the branch lengths are then updated for the estimated population size history and these steps are repeated until convergence. This was done for a default of five iterations (–num_iter 5).

### Alternative Methods for Selection Inference

To benchmark the performance of SIA for classification of sites under neutrality versus selective sweep, we ran the following methods: Tajima’s D (Tajima 1989), H1 (Garud et al. 2015), iHS (Voight et al. 2006), a summary statistics-based deep-learning model, and a tree-based statistic that is part of the Relate (Speidel et al. 2019) program. Tajima’s D, H1, and iHS were calculated with the *scikit-allel* package. Haplotypes of the entire 100 kb simulated genomic segment were used for Tajima’s D and H1 calculations. The unstandardized iHS was computed at every site with minor AF >5%, with respect to all other sites in the genomic segment (min_maf = 0.05, include_edges = True). iHS scores of all sites were then standardized in 50 AF bins. Finally, the iHS score of a genomic region was taken to be the mean of the iHS scores of all of its variant sites. For the summary statistics-based deep-learning model, we made use of the summary statistics used by S/HIC (Schrider and Kern 2016; Kern and Schrider 2018) as features for our deep-learning architecture. These included 11 sequence-based summary statistics (see Figure 3 in Schrider and Kern [2018]) which were used as features for our deep-learning model to distinguish among the two classes at hand (selective sweep vs. neutral drift). All statistics were collected along five consecutive 20-kb windows with the objective of identifying possible sweeps induced by a positively selected mutation in the third (middle) window. Some of these summary statistics corresponded to standard measures of diversity, such as ss (the number of segregating sites), π (Nei and Li 1979), Tajima’s D (Tajima 1989), θ_W_ (Watterson 1975), θ_H_ (Fay and Wu 2000), the number of distinct haplotypes (Messer and Petrov 2013), H1, H12, H2/H1 (Garud et al. 2015), Z_nS_ (Kelly 1997), and maximum value of ω (Kim and Nielsen 2004). For each of these statistics, we computed an average value for each of the five 20 kb windows for the simulated population. Finally, each summary statistic was normalized by dividing the value recorded for a given window by the sum of values across all five windows. The Relate tree-based selection test was performed with an add-on module (*DetectSelection.sh*) using the inferred genealogy with calibrated branch lengths at a site of interest, yielding a log_10_*P* value for each site.

We also compared the performance of SIA for selection coefficient inference with that of CLUES (Stern et al. 2019) and a genotype-based CNN framework (Flagel et al. 2019; Torada et al. 2019). Selection coefficient inference from true genealogies was performed with *clues-v0* (https://github.com/35ajstern/clues-v0, last accessed November 28, 2021). Transition probability matrices were built on a range of selection coefficients [0, 0.05] at increments of 0.0001 and present-day allele frequencies [0.01, 0.99] at increments of 0.01. Selection coefficient inference from Relate inferred genealogies was performed with CLUES (https://github.com/35ajstern/clues, last accessed November 28, 2021). Branch lengths of the genealogy at the site of interest were resampled with Relate for 600 MCMC iterations, and CLUES was run with the following arguments: “–tCutoff 10000 –burnin 100 –thin 5.” For the genotype-based CNN model, each simulated genomic segment was preprocessed by first sorting the haplotypes and then converting the segment to a fixed-size genotype matrix. Haplotype sorting was performed by 1) calculating the pairwise Manhattan distances between haplotypes; 2) setting the haplotype with the smallest total distance to all other haplotypes as the first haplotype; and 3) sorting the remaining haplotypes in increasing distance to the first haplotype. To convert the sorted haplotypes to a fixed-size genotype matrix, centered on the middle variant of a simulated region, up to 180 variants on each side were retained. Variants beyond 180 were discarded and if there were fewer than 180, the missing variants were padded with zeros. Ancestral and derived alleles were coded with 0s and 1s, respectively. Consequently, each simulated genomic region was encoded as a (198 × 360) binary matrix, along with a real-valued vector encoding the genomic positions of the variants in the matrix. The CNN model had a branched architecture––one branch with five 1D convolution layers taking the genotype matrix as input and another branch with a fully connected layer taking the vector of variant positions as input. The output of the two branches was flattened, concatenated and fed into three fully connected layers, followed by a linear output layer to predict selection coefficient (supplementary fig. S23, Supplementary Material online).

### Evaluation Metrics

To evaluate the performance of SIA’s classification model and alternative methods, we computed an ROC curve for the binary class at hand (“neutral” or “sweep”), to provide a more complete summary of the behavior of different types of errors. We further assessed the performance of SIA and alternative methods in terms of correctly predicting the selection coefficient numerically using mean absolute error (mae), root mean square error (rmse), coefficient of determination (*r*^2^), and visually using a box plot that compares the simulated ground truth against the predictions by the method at hand.

### Robustness Study

We carried out an extensive analysis of the robustness of our approach, considering not only alternative demographic parameters (such as population size), but also alternative parameters for recombination rate, mutation rate, time of selection onset, and selection coefficients. In all cases, we took care to test our prediction methods under parameters well outside the range used in training.

### Analysis of CEU Population in 1000 Genomes Data

We applied SIA to infer selection coefficients and AF trajectories in the 1000 Genomes (Auton et al. 2015) CEU population at 13 genomic loci with known association to phenotypes, some of which were previously identified as likely targets of positive selection (table 1). For each gene of interest, the ARG was inferred with Relate from SNPs within a 2-Mb window centered at the gene. Once the ARG was inferred, only SNPs with valid ancestral allele (“AA” INFO field in the vcf file) were retained for downstream analysis. Following the aforementioned protocol (see ARG Feature Extraction), features at all variant sites in the 2 Mb window above a derived AF threshold of 0.05 were extracted. Lastly, the SIA model was applied to classify neutrality versus selection, and infer selection coefficient and AF trajectory at each site.

### Localizing Sweeps in Southern Capuchino Seedeaters

We recently applied a combination of ARG inference and ML methods for identifying selective sweeps to study previously identified “islands of differentiation” in southern capuchino seedeaters and distinguish among possible evolutionary scenarios leading to their formation (Hejase, Salman-Minkov, et al. 2020). Taking advantage of its improved power and genomic resolution, we applied SIA to sequence data for the species for which we have the most samples, *S.hypoxantha*. We simulated training (250,000 neutral; 250,000 soft sweeps), validation (1000 neutral; 1000 soft sweeps), and testing (2,500 neutral; 2,500 soft sweeps) data sets for SIA under a demographic model inferred by G-PhoCS (Campagna et al. 2015). Simulations were performed using discoal with the following parameters: 1) mutation rate *μ* = 1e−9; 2) recombination rate *ρ* = 1e−9; 3) derived *N*_e_ = 130,000; 4) root divergence time = 1,850,000 generations ago; 5) root *N*_e_ = 1,450,000; 6) ancestral divergence time = 44,000 generations ago; 7) ancestral *N*_e_ = 14,380,000; 8) selection coefficient *s* ∼ *U*(0.001, 0.02); 9) initial frequency at which selection starts acting on the allele *f*_init_ ∼ *U*(0.01, 0.05); and 10) segregating frequency of the site under selection *f* ∼ *U*(0.25, 0.99). A total of 56 haploid sequences were sampled from each simulation, matching the number of *S. hypoxantha* individuals (28) in the real data. The SIA model for *S. hypoxantha* was built, trained and evaluated in an otherwise similar fashion to that for the CEU population as outlined above.

Using a subset of polymorphism data from Turbek et al. (2021) of 28 *S. hypoxantha* and 2 *S. minuta* individuals, we applied our trained model to localize selective sweeps in *S. hypoxantha* on 19 scaffolds that contain top *F*_ST_ peaks in at least one pairwise species comparison (Campagna et al. 2017) and/or harbor known pigmentation-related genes such as *ASIP* (located on scaffold 252; induces melanocytes to synthesize pheomelanin instead of eumelanin), *KITL* (located on scaffold 412; stimulates melanocyte proliferation), *SLC45A2* (located on scaffold 404; transports substances needed for melanin synthesis), and CAMK2D (located on scaffold 1717; cell communication), as well as 316 scaffolds that 1) are longer than 100 kb; 2) contain more than 1,000 variants; and 3) where more than 95% of sites have a consensus ancestral allele, as determined by four identical haplotypes for two individuals from the outgroup species *S. minuta*. The ARG was inferred with Relate for each scaffold independently. Once the ARG was inferred, the SIA model was applied to sites with consensus ancestral allele for classification and selection coefficient inference.

## Supplementary Material

Supplementary data are available at *Molecular Biology and Evolution* online.

## Supplementary Material

msab332_Supplementary_Data

## Data Availability

The simulation scripts and code for building and training the SIA model are publicly available on GitHub at github.com/CshlSiepelLab/arg-selection (last accessed November 28, 2021). No new data were generated for this study.
